# Supporting physical activity through co-production in people with severe mental ill health (SPACES): protocol for a randomised controlled feasibility trial

**DOI:** 10.1186/s40814-024-01460-0

**Published:** 2024-02-17

**Authors:** Gareth Jones, Laura Bailey, Rebecca J. Beeken, Samantha Brady, Cindy Cooper, Robert J. Copeland, Suzanne Crosland, Sam Dawson, Matthew Faires, Simon Gilbody, Holly Haynes, Andrew Hill, Emily Hillison, Michelle Horspool, Ellen Lee, Jinshuo Li, Katarzyna K. Machaczek, Steve Parrott, Helen Quirk, Brendon Stubbs, Garry A. Tew, Gemma Traviss-Turner, Emily Turton, Lauren Walker, Stephen Walters, Scott Weich, Ellie Wildbore, Emily Peckham

**Affiliations:** 1 Advanced Wellbeing Research Centre, Sheffield Hallam University, Sheffield, S9 3TU UK; 2 School of Medicine, University of Leeds, Leeds, LS2 9JT UK; 3 Department of Health Sciences, University of York, York, YO10 5DD UK; 4 School of Health and Related Research, University of Sheffield, Sheffield, S1 4DA UK; 5 Sheffield Health and Social Care NHS Foundation Trust, Distington House, Atlas Way, Sheffield, S4 7QQ UK; 6 Institute of Psychiatry, Psychology and Neuroscience, Kings College London, London, WC2R 2LS UK; 7 Institute for Health and Care Improvement, York St John University, York, YO31 7EX UK; 8 School of Health Sciences, City, University of London, London, EC1V 0HB UK; 9 School of Medical and Health Sciences, Bangor University, Gwynedd, LL57 2DG UK; 10 Sport and Physical Activity Research Centre, Health and Wellbeing Department, Sheffield Hallam University, Sheffield, UK

**Keywords:** Exercise, Severe Mental Illness, Health Behaviour, Pilot Studies

## Abstract

**Background:**

Severe mental ill health (SMI) includes schizophrenia, bipolar disorder and schizoaffective disorder and is associated with premature deaths when compared to people without SMI. Over 70% of those deaths are attributed to preventable health conditions, which have the potential to be positively affected by the adoption of healthy behaviours, such as physical activity. People with SMI are generally less active than those without and face unique barriers to being physically active. Physical activity interventions for those with SMI demonstrate promise, however, there are important questions remaining about the potential feasibility and acceptability of a physical activity intervention embedded within existing NHS pathways.

**Method:**

This is a two-arm multi-site randomised controlled feasibility trial, assessing the feasibility and acceptability of a co-produced physical activity intervention for a full-scale trial across geographically dispersed NHS mental health trusts in England. Participants will be randomly allocated via block, 1:1 randomisation, into either the intervention arm or the usual care arm. The usual care arm will continue to receive usual care throughout the trial, whilst the intervention arm will receive usual care plus the offer of a weekly, 18-week, physical activity intervention comprising walking and indoor activity sessions and community taster sessions. Another main component of the intervention includes one-to-one support. The primary outcome is to investigate the feasibility and acceptability of the intervention and to scale it up to a full-scale trial, using a short proforma provided to all intervention participants at follow-up, qualitative interviews with approximately 15 intervention participants and 5 interventions delivery staff, and data on intervention uptake, attendance, and attrition. Usual care data will also include recruitment and follow-up retention. Secondary outcome measures include physical activity and sedentary behaviours, body mass index, depression, anxiety, health-related quality of life, healthcare resource use, and adverse events. Outcome measures will be taken at baseline, three, and six-months post randomisation.

**Discussion:**

This study will determine if the physical activity intervention is feasible and acceptable to both participants receiving the intervention and NHS staff who deliver it. Results will inform the design of a larger randomised controlled trial assessing the clinical and cost effectiveness of the intervention.

**Trial registration:**

ISRCTN: ISRCTN83877229. Registered on 09.09.2022.

**Supplementary Information:**

The online version contains supplementary material available at 10.1186/s40814-024-01460-0.

## Introduction

### Background and rationale

People living with severe mental ill health (SMI) (e.g., schizophrenia, bipolar disorder and schizoaffective disorder) die on average 15–20 years prematurely [1], with over 70% of deaths attributed to preventable physical health conditions such as cardiovascular disease [2]. Whilst life expectancy in the general population has steadily increased over recent decades, life expectancy for people with SMI has declined [3]. Addressing this widening health inequality is a priority in the UK National Health Service (NHS) Long Term Plan [4]. Sedentary behaviour (any waking behaviour characterised by an energy expenditure ≤ 1.5 metabolic equivalents while in a sitting, reclining or lying posture) and physical inactivity (insufficient physical activity to meet physical activity guidelines) are amongst the leading causes of avoidable mortality and morbidity in the UK population [5]. The World Health Organization have stated that addressing these distinct behavioural targets is as important as encouraging smoking cessation [6]. Previous research has demonstrated that people with SMI engage in substantially lower levels of physical activity and higher levels of sedentary behaviour than those without SMI [3, 7]. Additionally, research has shown that people with SMI experience several unique barriers to engaging in physical activity such as increased mental health symptoms, lack of social support, and the side effects of medication [8].

Conventional treatment approaches for people with SMI tend to focus on pharmacotherapy and psychotherapy, and do not necessarily lead to improvements in physical health or quality of life (QoL) [9]. Obesity and inactivity remain important drivers of poor physical health, and behavioural approaches might be useful in mitigating the side effects of psychotropic medication such as weight gain.

In the wider population, there is robust evidence that higher levels of physical activity and lower levels of sedentary behaviour can reduce cardiovascular disease, diabetes and metabolic syndrome [5]. Previous small studies have suggested that physical activity can improve mental health symptoms in people with schizophrenia and major depression [10], but evidence about the acceptability, feasibility, and effectiveness of specific physical activity interventions is lacking. Additionally, a recent systematic review identified that current interventions have not been successful in engaging people with SMI in physical activity and identified the need for bespoke interventions to engage this population [7]. Consequently, interventions to promote physical activity and reduce sedentary behaviour are not routinely offered to people with SMI in NHS settings. Our work with service users has reinforced the aforementioned literature, illustrating that current physical activity programmes are not easily accessed by those with SMI.

Research shows the potential for physical activity interventions to normalise social activity and connect people with a mental illness to others ‘in the same boat’ [11]. In the Patient and Public Involvement and Engagement (PPIE) work that informed the SPACES (Supporting Physical Activity through Co-production in People with Severe Mental Ill Health) programme, people with SMI expressed a strong preference that such interventions should be provided within the healthcare system. People with SMI present with complex psychosocial needs that require clinical support; especially in the early stages of making behavioural changes. Staff supporting physical activity sessions outside clinical pathways might not be equipped with the skills and expertise to provide this input. Given the infancy of these approaches, previous research and PPIE consultation demonstrates that the provision of physical activity is most likely to work when offered within clinical pathways for individuals with complex psychiatric and social needs. Delivering a physical activity intervention within clinical pathways might also reduce the stigma associated with clinical environments for people with SMI. In addition, integration of physical activity with a community approach is consistent with established frameworks that emphasise connectedness, hope, identity, meaning in life, and empowerment [12]. This is not the same as delivering interventions outside a healthcare system. Thus, bespoke interventions to increase physical activity and improve QoL, mental health and physical health delivered through clinical pathways are urgently required in people with SMI. The SPACES programme aims to fulfil this aim.

The SPACES programme is split across four workstreams. Workstream one focused on co-production of a physical activity intervention for people with SMI embedded within the NHS [13]. The co-production process started before funding for SPACES was secured by using the perspectives of those with lived experience of SMI, carers and professionals who work with them to inform the conceptualisation of the SPACES programme. People with lived experience of SMI directed, reviewed, informed and refined the intervention in workstream one via focus groups. The focus groups were co-facilitated by SMI lived experience co-applicants. Additionally, PPIE work was embedded within the co-production model and led by the lived experience team members. The development of the intervention will be written up once the feasibility study is completed to allow any amendments to the intervention to be included in the publication describing the intervention development. An article explaining the SPACES co-production has been published [13]. Workstream two of the SPACES programme is a feasibility trial of the intervention developed in workstream one, the protocol for which is outlined in this paper. If feasibility is demonstrated, Workstream three will be a full-scale randomised controlled trial to determine the clinical- and cost-effectiveness of the intervention. Workstream four will be a process evaluation of the SPACES programme which will run concurrently with workstream three.

### Objectives

The main aim of this project is to explore the feasibility and acceptability of the SPACES intervention and the critical parameters for the design of a definitive randomised controlled trial (RCT). Objectives include:

1. To quantify the flow of participants (eligibility, recruitment and follow up rate) within the SPACES feasibility trial.
2. To evaluate proposed recruitment, assessment, outcome measures, and data collection methods within the SPACES feasibility trial.
3. To examine the delivery of the SPACES intervention (intervention uptake, retention and dose i.e., weekly, × 18 weeks).
4. To assess the acceptability of the intervention using a participant feedback form and interviews with participants and intervention providers.
5. To refine the SPACES intervention (intervention manual and training) for implementation by SPACES intervention delivery staff to people with SMI in community mental health trusts.
6. To use accelerometer-derived minutes per day of moderate-to-vigorous intensity physical activity (MVPA) to inform the sample size calculation for a full-scale trial.

### Trial design

Workstream two is a two-arm randomised controlled feasibility trial. Eligible participants will be randomised into one of two study arms: usual care plus intervention aimed at increasing physical activity or usual care alone. Outcome measures will be collected at baseline, three and six months. The trial was registered with the ISRCTN registry (ISRCTN83877229. Registered on 09.09.2022).

## Methods: participants, interventions, and outcomes

### Study setting

The study will be conducted across 4–7 geographically dispersed NHS mental health trusts in England.

### Eligibility criteria

#### Inclusion criteria

Patients will be eligible to join the study if they meet the following inclusion criteria:

1. Age: 18 years or over.
2. A primary ICD-10 or DSM-V diagnosis of SMI (schizophrenia, delusional/psychotic illness or bipolar disorder) as documented in General Practitioner (GP) or psychiatric notes.
3. Able to walk unaided.

#### Exclusion criteria

Patients will not be eligible for the study if they meet one or more of the exclusion criteria:

1. People who lack capacity to participate in the trial as guided by the Mental Capacity Act (2005) [14].
2. Primary diagnosis of drug or alcohol abuse.
3. Medical contraindication to physical activity as ascertained by GP or mental health team.
4. Already physically active (e.g., > 300 min/week of self-reported MVPA).
5. Unwilling to wear accelerometer.
6. Non-English speakers.

### Who will take informed consent?

Potential participants will have the opportunity to discuss involvement in the trial with a local NHS site researcher. If the participant is still interested in taking part in the study, written informed consent will be taken. Informed consent will preferably be collected during a face-to-face meeting with the participant, however, consent can also be taken over the phone if preferred, where the researcher will read out the consent form and complete it on behalf of the participant. A copy of the completed consent form will be sent to the participant.

### Interventions

#### Explanation for the choice of comparators

This trial has two arms, intervention and usual care. The definition of usual care is ‘the wide range of care that is provided in a community whether it is adequate or not, without a normative judgement’ [15]. Participants in both the intervention and usual care arms will continue to be able to access their usual care from primary care, secondary care, community, and social services. Having the ability to compare the intervention arm with the usual care arm enables the study to understand any differences between people with SMI receiving or not receiving the intervention in relation to the study outcomes.

#### Intervention description

The SPACES intervention aims to support people with SMI to initiate and maintain physical activity. The intervention comprises two core components, an 18-week group-based physical activity component and a one-to-one consultation component, both of which are facilitated by a physical activity coordinator (PAC: NHS staff with suitable experience to deliver the intervention). Suitable PAC experience will include previous history of delivering physical activity interventions to those with SMI and relevant physical activity delivery training and qualifications. The intervention will last up to 20 weeks (one-to-one consultations are offered prior to the physical activity component), with participants being offered up to 18 group physical activity sessions, up to 10 one-to-one sessions consisting of 3–6 long form consultations (30–60 min) and 2–4 check-ins (15–30 min). The intervention group will include between 6–12 participants and be supported by two PACs (four to six are trained at each NHS site for programme continuity e.g., covering annual leave). All PACs will receive training and a PAC manual on how to deliver the intervention to aid consistency. PACs will also receive ongoing bi-weekly support from the SPACES central team during the intervention delivery.

The weekly two-hour group sessions will comprise of three components, 1) up to 60 min physical activity, 2) 30-min themed discussion and 3) 30 min social time. The physical activity component will rotate between outdoor walking, indoor activity classes, and community taster sessions. Weeks 2–17 are recommended to include six outdoor walks, six indoor activity classes and four community taster sessions. Weeks one and eighteen are gentler in physical activity with a greater focus on the social component, and do not include a themed discussion. All physical activity sessions will be graded depending on the needs of the participants (e.g., resistance, balance, cardiovascular exercises) and will gradually progress (e.g., intensity or duration) over the intervention with a focus on physical activity of at least moderate intensity. The themed discussions will be guided by the PACs and cover relevant topics, including ‘getting started/keeping going with physical activity’, ‘benefits of increasing physical activity and reducing sedentary behaviour’, ‘overcoming barriers/hurdles to doing physical activity’, ‘being active in everyday life’, ‘staying motivated’, ‘keeping energised’, ‘exploring local indoor activities’ and ‘exploring local outdoor activities’. Each of the eight topics will be repeated across the intervention period. The time for informal socialising will be facilitated by a PAC, but not led by them. Participants will be encouraged to attend the whole session, however it is entirely up to them if they do not wish to attend one or more of the components. The session will be delivered in a suitable (e.g., adequate room size, location) NHS or community venue across the 18 weeks.

Alongside the weekly sessions, participants will be offered one-to-one long-form consultations at entry, mid and exit points to the intervention, and shorter check-ins in-between (i.e., between entry and mid, and mid and exit long-form consultations). At entry consultation(s), PACs will discuss topics such as participant physical activity, undertake goal setting, and explore any concerns of specific barriers/needs the participant may have. This session will be offered prior to the first group-based physical activity session. Mid-point consultation(s) will discuss topics such as maintaining motivation, overcoming obstacles, and goal setting. Exit consultation(s) will include discussion topics such as action and coping planning, goal setting and strategies to maintain motivation post trial. One-to-one consultations will be delivered face-to-face, over the phone, or by video call according to participant preference. Video sessions will use approved systems (such as NHS ‘Attend Anywhere’ platform). Face-to-face sessions will be delivered in the participant’s home or at an NHS or community venue according to participant preference.

Participants will also be offered additional intervention components. Firstly, the option of self-monitoring using either a self-complete physical activity diary or a wearable (such as a pedometer). If a participant wishes to use a wearable and they do not personally own one, then a device will be provided by the study team. Secondly, a participant handbook will be provided, specifying study information, relevant contact details of NHS intervention staff, information about physical activity such as Chief Medical Officer physical activity guidelines [16], and tasks designed to engage and support participants through their physical activity journey through the intervention. Thirdly, participants will be offered attendance prompts (e.g., brief telephone reminders) for the group physical activity sessions and one-to-one consultations. Fourthly, social support will be offered to participants through both peer (via the group sessions) and professional (via the PACs) support. Participants in the intervention group will continue to receive their usual care / treatment alongside the physical activity intervention and no treatment will be withheld.

The intervention was created using a series of inputs, including previous literature knowledge [17] and relevant expertise in intervention design, a series of focus and consensus groups, psychological theory (e.g., self-determination theory [18]) and the Behaviour Change Technique taxonomy [19], and evidence from a physical activity questionnaire for people with SMI [20]. These inputs were guided by co-production [13], which included people with SMI, professionals who support those with SMI, and academic experts. The final intervention included 14 behaviour change technique themes and 30 individual techniques from the behaviour change technique tool [21] (see supplementary material ‘Intervention BCT IDs’). The participant handbook and PAC manual are available upon request.

#### Criteria for discontinuing or modifying allocated interventions

There are no specific criteria for discontinuing or modifying the intervention, however, there will be some flexibility in the intervention as PACs will utilise Rating of Perceived Exertion (RPE) scales [22] to enable participants to exercise at an appropriate intensity for themselves. To facilitate this further, PACs will be provided with possible modifications of the indoor exercise class exercises to increase or decrease exercise intensity. Participants can choose to withdraw from the intervention for any reason, or if they are advised by their medical practitioner to do so due to health reasons.

#### Strategies to improve adherence to interventions

Intervention adherence will be monitored by a combination of notes recorded by the PAC and the use of participant logs to estimate the session duration and number of sessions attended by each participant. In addition, a selection of intervention delivery sessions across sites will be observed by a member of the SPACES research team. Participants who miss two consecutive physical activity sessions will be contacted by a PAC, in an effort to aid adherence.

#### Relevant concomitant care permitted or prohibited during the trial

Participants will receive usual care throughout the trial. There are no prohibited factors to trial participation.

#### Provisions for post-trial care

There is no post trial care planned.

### Outcomes

Participants will be asked to complete outcome assessments at baseline, three and six months. See Table 1 for assessments by study timepoint.

**Table 1:** Participant assessment and time point requirements throughout the study

Assessment	Time required	Timeline (months post randomisation)				
		Baseline	Randomisation		3-month Follow-up	6-month Follow-up
**Eligibility and consent**						
Eligibility	NA	X				
Consent	15 min	X				
Randomisation			X			
**Background**						
Relevant contact details	5 min	X				
Body Mass index	5 min	X			X	X
**Mental health details**						
Mental health history		X				
Current mental health status		X			X	X
Current medications		X			X	X
Referrals to mental health services		X			X	X
Admissions to hospital related to mental health		X			X	X
**Physical Activity**						
Accelerometer data collection	10 days	X			X	X
Adverse event reporting		Ongoing collection				
**Questionnaires**						
Acceptability Intervention Measure (AIM) (for intervention participants only)	20–30 min total					X
Patient Health Questionnaire (PHQ-9)		X			X	X
Generalised Anxiety Disorder-7 (GAD-7)		X			X	X
Short Form-12 (SF-12)		X			X	X
Simple Physical Activity Questionnaire (SIMPAQ)		X			X	X
EuroQol-5D-5L		X			X	X
Recovering Quality of Life (ReQol)		X			X	X
Healthcare resource use		X			X	X
**Interviews**						
Follow-up interview (*n* = 20)	30–60 min					X

### Primary outcome measure

The main objectives of this feasibility study are to explore the acceptability of the physical activity intervention, and the central parameters for the design of a full-scale trial (e.g., recruitment, retention, outcome completion, and intervention adherence).

The acceptability of the intervention will be investigated using a short proforma provided to all intervention participants at follow-up which includes the acceptability of intervention measure (AIM; [23]), qualitative interviews with approximately 15 intervention participants and 5 people who have delivered the intervention, and data on intervention uptake, attendance, and attrition. Acceptability data will be analysed by the central SPACES team to determine acceptability of the intervention and amended accordingly to improve intervention acceptance. The feasibility of this pilot trial and the potential for undertaking a future large-scale main trial will be measured primarily using participant recruitment data.

### Secondary efficacy outcome measures

Secondary outcome measures include body mass index (weight (kg) / [height (m)]^2^), depression (PHQ-9; [24]), anxiety (GAD-7; [25]), physical activity and sedentary behaviours (accelerometer-assessed metrics & SIMPAQ; [26]), health-related quality of life (EQ5D [27], SF-12 [28], REQoL [29]), healthcare resource ​​use collected via a bespoke questionnaire designed for SPACES (see Table 1), and adverse events.

### Accelerometer details

An accelerometer measures movement of an object (e.g., a person). Accelerometers designed to measure a person’s physical activity are often wrist worn and resemble a watch. They measure the volume and intensity in which someone moves within a period of time. Accelerometer devices can also measure parameters such as sleep and sedentary behaviour. A more detailed breakdown of the objective accelerometer data analysis includes reporting sedentary behaviour, light intensity activity, moderate intensity activity, vigorous intensity activity, and MVPA using mean time per day over all available valid days, and sleep window duration. Accelerometer will be worn for a measurement period of 10 days, starting at 12am the day after a participant clinic visit. Idle sleep mode, wireless, heart rate and IMU will all be disabled. ActiLife Lite (ActiGraph accelerometer) and OMGUI (Axivity AX6 accelerometer) software will be used by SPACES site researchers to download data from the accelerometers after each 10-day wear period before secure transfer to the central study team. Data will be further processed using GGIR v2.4–0 [30] in R [http://cran.r-project.org].

### Participant timeline

**Fig. 1:**
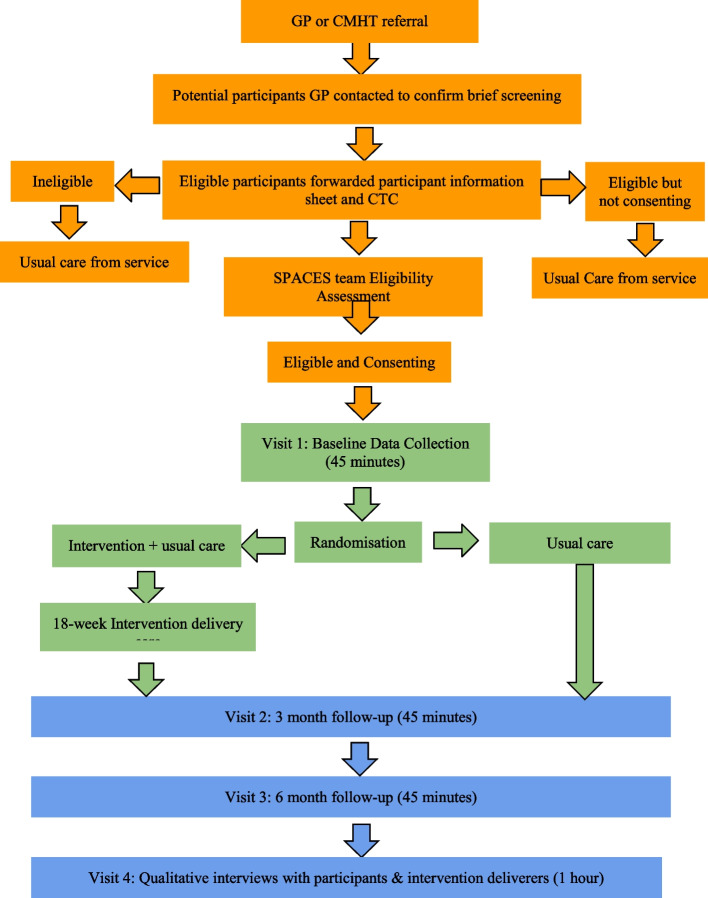
Flow diagram of a participant journey through the SPACES feasibility trial

The participant schedule through the study, including eligibility, consent, randomisation, baseline and follow-up assessment are highlighted in Fig. 1.

### Sample size

The sample size for a feasibility study should be adequate to estimate the uncertain critical parameters (SD for continuous outcomes; consent/recruitment rates, attrition, intraclass correlation coefficient (ICC)) needed to inform the design of the full RCT with sufficient precision. As a feasibility study we propose to recruit for a fixed time (T) and not a sample size (N). We propose to recruit for 6 months at between 4 and 7 sites (e.g.: 4 × 6 = 24 site*months of recruitment). We believe that in order for the main trial to be feasible we need to recruit enough participants to run a full SPACES intervention group in the intervention arm at each site (recruiting at least 2*6 = 12 participants) which would be a rate of 12/6 = 2 participants per site per month. There are several flat rules of thumb for sample sizes for two-armed external pilot/feasibility trials ranging from 24 in total [31], to 30 [30, 32], to 50 or 60 [33], and 100 [34] and recruiting for 24 site*months should enable us to exceed these ranges (of 48 to 140 participants).

Interviews will be conducted with 15 participants from the intervention group and 5 intervention delivery staff members (see supplementary material for the topic guide). This sample size will be continuously evaluated during data collection process’ to ensure adequate qualitative sample size information power is met [35]. A combination of purposive and convenience sampling will be utilised to ensure adequate and varied sampling across all involved NHS sites (2–4 participants and × 1 PAC per site) from the cohort of participants and delivery staff available.

### Recruitment

#### Recruitment of NHS sites

Sites were recruited to the SPACES programme via email invitation. Emails were sent to all mental health NHS Trust teams who had provided a letter of support for the SPACES grant application, with details of the SPACES feasibility trial. In addition, mental health NHS Trust teams with a working relationship to the research team were also approached as potential sites. Seven trusts responded to the call, at which point further information was provided regarding the trial and clarification was offered to any questions trusts had about the trial. One site was lost during this process, leaving six trusts to participate in the SPACES feasibility trial.

### Recruitment of participants

Participants will be identified via secondary care through Community mental health teams (CMHT) or via primary care practices. General Practitioner (GP) surgeries and CMHTs will be asked to consult their patient lists to screen against the inclusion and exclusion criteria (see eligibility criteria section) for potentially eligible participants. Participants with a documented diagnosis of SMI are eligible to participate regardless of whether they are under the care of a mental health NHS trust or not. Participants identified as potentially suitable for the SPACES trial will be provided with a copy of the patient information pack via the post or by their GP/ CMHT directly. The patient information pack will contain an invitation letter, participant information sheet, a consent to contact (CTC) form, and a pre-paid return envelope. If interested, the potential participant will either return the CTC form to the SPACES research team in the pre-paid envelope or the recruiting clinician will complete a verbal CTC form on behalf of the potential participant and return it to the SPACES study team by email, post or pass the details to a SPACES researcher over the phone. In addition, members of CMHTs and GPs or practice nurses will be invited to directly refer eligible patients to the SPACES team during any encounter they have with the potential participant (e.g., face to face or telephone appointments). CMHT/ GP surgeries members will be provided with patient information packs to give to service users who are receptive to participating in the SPACES trial. The pack will either be provided directly at a face-to-face meeting, electronically, or via the post if the meeting was over the phone. CMHT members, GPs or practice nurses can gain verbal CTC from potential participants during these encounters and pass on their information to the SPACES team if verbal CTC is provided.

Posters and flyers advertising the study will also be placed in centres which are recruiting to the study. In addition, posters may also be displayed on participating trust’s Facebook pages and Twitter accounts. Patients interested in taking part in the study will be asked to approach their care coordinator for further information about the study, and if eligible and appropriate will be given a patient information pack.

On receipt of a completed CTC, a SPACES researcher will contact the potential participant to discuss the study and provide an opportunity to answer any questions and, if appropriate, arrange for consent to be taken if they are happy to participate given the study information provided.

To ensure the suitability of people prior to recruitment, the GPs or CMHTs of each potential participant will check they meet the eligibility criteria. This will be done prior to the potential participant being approached by the SPACES research team.

### Assignment of interventions: allocation

#### Sequence generation

Allocation will be through block, 1:1 randomisation (with random permuted blocks of various integer multiple of two sizes) with a separate schedule for each site. There will be no additional stratification factors, beside site, in the randomisation sequence. The study statistician in Sheffield CTRU will generate the randomisation schedule prior to the start of the study using its own web-based in-house CTRU (SCRAM) randomisation system.

#### Concealment mechanism

Allocation concealment will be ensured; the web-based system is secure and will not release the randomisation code until the patient has been recruited into the trial, which takes place after all baseline measurements have been completed. The sequence generation is conducted by the study statistician who is not involved in randomising and details of block sizes will not be communicated to the study team.

#### Implementation

The study statistician will generate an allocation sequence. At the second baseline data collection point (accelerometer return), the SPACES researcher will contact the SPACES central team to be provided with the participant’s randomisation assignment. Participants are then told their allocation.

### Assignment of interventions: blinding

#### Who will be blinded

By the nature of the interventions used within this study, blinding of the participants, clinicians and the researchers is not possible. However, those involved in the analysis of the data will be blind to group allocation whilst the trial is ongoing and until the analysis is complete.

#### Procedure for unblinding if needed

Study personnel who are responsible for analysing data and reporting results will remain blinded throughout the study period. As clinicians and researchers are not blinded to allocation there will not be a need for unblinding.

### Data collection and management

#### Plans for assessment and collection of outcomes

Data collection will take place across three time points; baseline, and three- and six-months post-randomisation. Participant 1:1 randomisation occurs immediately after baseline data collection is complete. At baseline only, demographic and medical history information will be collected. At baseline, and the 3- and 6-month follow-ups, primary and secondary outcome data will be collected by a SPACES researcher at a face-to-face meeting in NHS premises, the participant’s home or another mutually convenient location. If for any reason a participant is not able to meet face-to-face, where possible data will be collected over the phone, by postal questionnaire or via an online questionnaire. See data collection schedule for more details (Table 1).

Physical activity and sedentary behaviour data will be gathered using Actigraph GT9X Link or Axivity AX6 accelerometers.^1^ Participants will be asked to wear the accelerometer for 10 days prior to each data collection point (baseline, three- and six-month follow-up). Accelerometers will be provided and collected from participants at each data collection point.

#### Plans to promote participant retention and complete follow-up

The following plans will be utilised in order to promote participant retention throughout the trial period:

- Regular communication with study participants will be provided throughout the study, with participants being provided with NHS site SPACES team contact details for any potential queries.
- If a questionnaire is returned incomplete or contains errors, a researcher will call the participant for clarification or completion of missing data.
- Participants will be advised that they are able to contact a member of the research team if they require assistance with completing a questionnaire.
- Participants will be asked to provide contact details of up to three people who can be contacted if the SPACES team are unable to contact the participant directly.

In order to understand the feasibility of the trial, study withdrawal and dropout rates will be monitored and recorded. Participants will be informed that they may withdraw from the study at any time without influencing their future care or treatment. When a participant wishes to withdraw from the study, we will confirm the withdrawal type from one of the following possible options:

- Where a participant in the intervention arm of the study wishes to withdraw from the study intervention, but is prepared to continue completing follow-up questionnaires (i.e., no further intervention sessions are attended but follow up data is still collected). This is classed as ‘Withdrawal from the intervention’.
- Where a participant in the intervention arm of the study wishes to withdraw from completing any further follow-up data but wishes to continue with the intervention This is classed as ‘Withdrawal from follow-up’.
- Where a participant in the intervention arm wishes to withdraw from both the study intervention AND from completing any further follow-up data collection. This is classed as ‘Full withdrawal’. Where a participant in the usual care arm wishes to withdraw from follow up this is classed as full withdrawal.

### Data management

Data management will be provided by University of Sheffield Clinical Trials Research Unit (CTRU) who adhere to their own Standard Operating Procedures (SOPs) relating to all aspects of data management, including data protection and archiving. The study will use the CTRU's in-house data management system (Prospect) for the capture and storage of study-specific participant data. Access to Prospect is controlled by usernames and encrypted passwords. Designated staff at each site will enter data from source documents into the Prospect database. Once data have been entered, electronic validation rules are applied to the data on a regular basis; discrepancies are tracked and resolved through the database. All collection, storage, processing and disclosure of personal information will be performed in compliance with the GDPR & Data Protection Act 2018.

### Confidentiality

Participants will be informed of their right to confidentiality. All information collected during the study will be kept strictly confidential. Information will be held securely in paper and/or electronic formats at either the Sheffield CTRU, University of Sheffield, or Department of Health Sciences, University of York. All participants will be anonymised at the point of consent, by assignment of a study number. This will ensure that all personal data collected for the study are anonymous. Personal data and anonymised data will be stored separately in a restricted access folder on a secure university server, access will be password protected, and restricted to members of the SPACES research team. Quotations from participants may be used in research reports and other publications and presentations; however, care will be taken to protect the anonymity of participants so that others are not able to identify them.

### Statistical methods

#### Statistical methods for primary and secondary outcomes

As the trial is a pragmatic parallel group RCT design the statistical analysis will be performed on an intention-to-treat-basis and will be reported and presented according to the CONSORT statement extension to feasibility trials [36]. As a feasibility study the analysis will be mainly descriptive and focus on confidence interval estimation and not formal hypothesis testing.

We will use the data from this feasibility study, including reported rates of consent, recruitment and follow-up, and outcome measures, accelerometer-assessed minutes of MVPA per day (averaged over all available valid days), accelerometer-assessed total physical activity and minutes per day of sedentary behaviour (averaged over all available valid days), BMI, AIM, PHQ-9, SF-12, SIMPAQ, EQ-5D-5L, ReQoL to summarise overall and by randomised group, to inform sample size estimation for the main trial.

Accelerometer parameters will include daily 16-h per day wear time to constitute as valid wear time, intensity gradient cut off points of < 30 mg, 30–100 mg, 101–400 mg and > 400 mg (inactive, light, moderate and vigorous activity respectively). Mean MVPA per day is calculated on 5-s epochs with a 1-min bout duration, inclusion criteria of > 80%. All days (≥ 4 valid days), weekends (≥ 1 valid days) and weekdays (≥ 3 valid days) will be calculated. Mean acceleration and intensity gradients will also be reported. ActiGraph GT9X link [37] and Axivity AX6^1^ [38] accelerometers will utilise 100-Hz measurement frequency rates. Accelerometer data will be processed using GGIR v2.4–0 [39] in R [http:/cran.r-project.org].

We will also include, as part of the feasibility analysis, estimation of the effect size for accelerometer-determined physical activity 6-months post-randomisation (the probable primary endpoint for the definitive study), defined as the mean (averaged over all available valid days) minutes of MVPA per day, with confidence interval estimates to check that the likely effect is within a clinically relevant range (as confirmation that it is worth progressing with the full trial (i.e. 95% CI includes a difference of 6 min of MVPA per day)). For this we will use a partially clustered mixed effects model with 6-month minutes of MVPA as the outcome and baseline minutes of MVPA, site and randomised group as fixed effects and SPACES intervention groups as random effects. The ICC from this model will be reported and will inform the ICC estimate for the main trial. This information along with the acceptability of the study design and protocol to patients/GPs; the safety of the intervention; adherence to the intervention, patient recruitment and attrition/retention rates will enable us to determine whether or not the definitive RCT is feasible, within a satisfactory timescale and cost envelope using UK sites alone.

We will estimate the intervention costs based on the delivery of the related activities and the study records over the intervention period. We will also test the feasibility and acceptability of the bespoke health care and service use questionnaire. Analysis of the completeness of participants’ responses and use of listed services together with additional services recorded by participants, will enable further revision to collect most relevant data in the full RCT and eliminate questions with few responses. The completeness and descriptive statistics of healthcare resources and EQ-5D-5L will be summarised by participants’ allocated study arm. No formal cost-effectiveness analysis will be undertaken as the feasibility study is not sufficiently powered.

#### Interim analyses

No interim analyses are planned for this trial.

#### Methods for additional analyses (e.g., subgroup analyses)

Qualitative data will be analysed utilising an inductive approach and thematic analysis [40] to explore both participants and intervention delivery team experiences of the SPACES intervention. Participant data will be categorised to understand different perspectives. Qualitative analysis software, NVivo, will be used to process and analyse the data. Feedback from both participants and PACs will inform refinement of the intervention, including the intervention manual and training, with the aim to improve the acceptability and feasibility of the SPACES intervention.

Critical variables to enable success in the full RCT (workstream 3) will follow a traffic light system with recruitment of 100%, i.e., an average of 2 participants per centre per month being green, 50–99% (1 – 1.9 participants per month) being amber, and below 50% being red. This will enable us to select the correct number of centres for the full RCT. We will also test our ability to achieve follow-up at our target rate in the feasibility study, and to ensure there is equal retention between arms.

#### Methods in analysis to handle protocol non-adherence and any statistical methods to handle missing data

We will analyse the data on an intention to treat basis, whereby all participants recruited in the study will be entered into the analysis, regardless of whether or not they dropped out or partially adhered to their treatment method.

The planned primary outcome measure for the future trial, minutes of MVPA at 6 months, will be analysed at in a multiple linear regression model to compare the intervention versus usual care.

#### Plans to give access to the full protocol, participant-level data, and statistical code

This paper provides the full protocol. Interested individuals should contact the study co-Chief Investigator (EP) if interested in other data or documentation of the study.

### Oversight and monitoring

#### Composition of the coordinating centre and trial steering committee

A programme management group comprising the co-Chief Investigators, co-applicants, the programme manager, the trial coordinator, the statistician and researchers from each of the research sites who will meet quarterly to oversee the general running of the project and all its components such as the progress of the trial and review recruitment. The trial will be managed on a day-to-day basis by the Programme Manager supported by the Chief Investigators and Sheffield CTRU team. The group will provide timely reports on the progress, or completion, termination or discontinuation of the study to the ethics committees.

The trial will be overseen by an independent Programme Steering Committee (PSC) consisting of the Chief Investigators of the study, an independent chair and at least two other independent members (including someone with lived experience). The PSC will meet on at least a six-monthly basis via face-to-face meetings or an online platform to discuss progress of the trial, or more often as appropriate. The Data Monitoring and Ethics Committee (DMEC), research team and statisticians will report to the PSC as necessary.

#### Composition of the data monitoring committee, its role, and reporting structure

The committee will consist of independent experts, including a statistician and mental health professionals, who are independent of the principal investigator and the study team. Its remit will be to monitor the trial data, in particular quality control and quality assurance of the data collected, and progress of the trial including adherence to the trial protocol. The committee will also examine and ensure that the dignity, rights, safety and wellbeing of all study participants are maintained at all stages of the trial. Data reports will be supplied, including any adverse events, and the committee will have access to summary data and documentation. The Chair of the DMEC will be informed of any adverse events that arise from the study or regarding participants during the study period, and they will be in a position to recommend suspension or ending the trial depending on the severity of the adverse event.

#### Adverse event reporting and harms

Data on adverse events will be collected on a case-by-case basis. All adverse events and serious adverse events will be evaluated by an independent reviewer and the Chief Investigator within 24 h of the event being reported. A summary table of adverse events will be compiled on an ongoing basis and will be provided to the DMEC at DMEC meetings. The summary will include a description of the adverse event, the date of onset, severity, action taken, outcome, whether the adverse event was expected or not, and an evaluation of the relationship of the adverse event to study procedures. Adverse event reports and summaries will not include any patient identifiable data and participants will be identified only by study ID number. Example adverse events include, but are not limited to:

- Muscle soreness which fails to resolve 72 h following a physical activity session
- Substantial increases in pre-existing pain which fails to resolve 72 h following a physical activity session
- Development of new musculoskeletal pain which fails to resolve 72 h following a physical activity session
- Injuries sustained as a result of an accident incurred travelling to or from a physical activity session

### Frequency and plans for auditing trial conduct

To review study conduct, meetings with the independent programme steering committee will be held six monthly. In addition, programme management team meetings will be held monthly with key stakeholders.

### Plans for communicating important protocol amendments to relevant parties (e.g., trial participants, ethical committees)

Protocol changes that impact study procedures or risk to human subjects will be approved by the West of Scotland NHS Research Ethics Committee and if applicable reflected in a revised participant information sheet and consent form which will be reviewed and signed by all active participants. Protocol changes will also be reported to NIHR PGfAR in progress reports.

#### Dissemination plans

Findings from this study will be shared publicly and disseminated through publication in peer-reviewed journals, conference presentations and social media outlets.

## Discussion

This protocol outlines the methods to be used in the SPACES feasibility and acceptability trial. This study outlines step two (assessment of intervention feasibility and evaluation design) or four, of the NIHR’s updated framework on developing complex interventions and considers the six core elements for intervention design [41]. We know that people living with SMI, on average, die 15–20 years prematurely [1], with over 70% of these deaths being attributable to preventable physical health conditions [2] linked to health behaviours such as physical (in) activity and sedentary behaviour. Furthermore, research demonstrates that people with SMI are less active than the general population [3, 7] and experience unique barriers to being physically active [8]. This study, therefore, aims to address an important health need, with an opportunity to manifest positive strategies to reduce the widening health inequality gap between those with SMI and the general population. Conventional treatment methods to reduce this gap have not necessarily been successful in improving physical health or QoL [9] for those with SMI. Previous studies attempting to address physical activity behaviour for the SMI population have had various limitations, such as sample size, however, have demonstrated promise for behaviour change [17]. In addition, the feasibility and acceptability of a physical activity intervention delivered within NHS pathways is yet to be understood.

Through assessing the feasible and acceptable of the SPACES intervention, we will uncover the necessary information to improve and refine the intervention and implementation process. This knowledge will be used to inform a larger scale RCT, investigating the effectiveness of the SPACES intervention for various health outcomes. Understanding effective methods to enable people with SMI to live healthier, more active lives, is likely to have a positive impact on the widening health inequalities gap between those with SMI and those without SMI. The SPACES intervention was co-produced by those with lived experiences of SMI and professionals who support those with SMI, thus, is well-positioned to reveal potentially effective strategies to support those with SMI to live physically active lives.

## Trial status

Trial recruitment commenced on 01st December 2022 and was completed by 20th April 2023. The trial closed with all follow-ups complete on the 18th December 2023. Current protocol version is 1.2 (dated 12/02/2024). 

### Supplementary Information

**Additional file 1: Appendix A**

## Data Availability

Any data required to support the protocol will be supplied on request.

## Abbreviations

AIM: Acceptability Intervention Measure
BMI: Body Mass Index
CI: Confidence Interval
CMHT: Community Mental Health Team
CONSORT: Consolidated Standards of Reporting Trials
CTC: Consent to Contact
CTRU: Clinical Trials Research Unit
DMEC: Data Monitoring and Ethics Committee
DSM-V: Diagnostic and Statistical Manual of Mental Disorders – 5
EQ5D: Euro Quality of Life 5 Dimensions
GAD: Generalised Anxiety Disorder assessment
GP: General Practitioner
Hz: Hertz
ICD: International Classification of Diseases
ISRCTN: International Standard Randomised Controlled Trial Number
MVPA: Moderate to Vigorous Physical Activity
NIHR: National Institute for Health and Care Research
NHS: National Health Service
PA: Physical Activity
PAC: Physical Activity Coordinator
PGfAR: Programme Grants for Applied Research
PHQ: Patient Health Questionnaire
PSC: Programme Steering Committee
QoL: Quality of Life
RCT: Randomised Controlled Trial
ReQol: Recovering Quality of Life
RPE: Rate of Perceived Exertion
SF-12: Short Form-12
SIMPAQ: Simple Physical Activity Questionnaire
SMI: Severe Mental Ill Health
SOP: Standard Operating Procedure
UK: United Kingdom

## Footnotes

1 Axivity AX6 accelerometers are proposed to be used with one NHS site once the minimum recruitment threshold has been achieved. The ActiGraph accelerometers battery life did not consistently reach 10 days recording at the specifications (e.g. 100 Hz) used in this study during testing (9-10 days were observed). The Axivity AX6 accelerometers at the given specifications do manage 10 + days of data collection, thus using Axivity AX6s at one site in this feasibility trial will enable us to compare the utility and acceptability of the Axivity accelerometers alongside the ActiGraphs. It is envisaged that due to the battery life favouring the Axivity accelerometers, these will be used in the main trial if deemed acceptable.
